# Prognosis, treatment decision-making and value: A qualitative exploration of the emerging role of breast cancer prognostic assays

**DOI:** 10.1371/journal.pone.0322509

**Published:** 2025-07-18

**Authors:** Gillian Parker, Stuart Hogarth, Jennifer Fishman, Fiona A. Miller

**Affiliations:** 1 Institute of Health Policy, Management and Evaluation, University of Toronto, Toronto, ON, Canada; 2 Department of Sociology, University of Cambridge, Cambridge, United Kingdom; 3 Department of Equity, Ethics, and Policy, McGill University, Montreal, QC, Canada; Beth Israel Deaconess Medical Center, UNITED STATES OF AMERICA

## Abstract

**Background:**

Breast cancer prognostic assays are emerging as tools used by physicians in the cancer treatment decision-making process. This technology is new, and we must interrogate the integration of these assays into clinical practice and their effect on prognosis and treatment for both providers and patients. The objective of this study was to explore perspectives on the use and integration of breast cancer prognostic assays in clinical care.

**Methods:**

15 international researcher-physician/scientist key opinion leaders who had conducted studies on breast cancer prognostic assays were interviewed. Participants had conducted studies using five different assays. The interview guide was developed through a literature review and leveraged extensive data collected on key clinical utility outcomes for the assays. All interviews were conducted virtually, recorded, and transcribed verbatim. Data were analysed using thematic analysis.

**Results:**

Three novel themes emerged from participant’s perspectives on the use and value of these assays. The emerging role of prognostic assays to identify overtreatment and unnecessary care was highlighted by the majority of participants. The primary value of these tools is to identify patients who will not benefit from adjuvant chemotherapy. Participants reported that current standard practice is to overtreat and portrayed the binary or definitive results of these assays as an important tool to reduce overtreatment. Participants also provided insights into deliberate efforts to integrate the assays into clinical practice and how improved quality of life and reduction in overtreatment was positioned to justify high cost of the assay. Finally, participants reported how the perspectives and uses of these assays vary significantly in different countries and cultures. This jurisdictional variation in cancer prognosis and treatment was observed as producing uneven and sometimes problematic interpretations of value for the assays.

**Conclusions:**

The results of this study provide insights into the integration of prognostic assays into healthcare services. The assays are seeking to extend the boundaries of their clinical utility through identifying overtreatment and low value care. Efforts to integrate these assays and justify their high prices are unpacked and reveal complex and contradictory factors. Finally, these results illuminate that the varied approaches to cancer treatment, and varied use of chemotherapy create disparate perceptions of value for the assays.

## Background

Claims that genomic and biomarker technologies will transform healthcare have been central to visions of a new era of personalised or precision medicine. However, the advent of prognostic assays in clinical care has coincided with growing regulatory scrutiny of new diagnostic technologies [1]. Thus it is perhaps unsurprising that the scale and pace of the genomic revolution has been less dramatic than promised, and that some of the most hyped applications, such as polygenic risk scores and pharmacogenetic testing, have failed to convince sceptics. Much of the debate about these technologies has centred on issues of what is increasingly referred to as clinical utility, a concept that was introduced two decades ago as part of a broader framework for the evaluation of genetic tests [2]. Clinical utility potentially encompasses clinical, comparative and cost effectiveness and its potential breadth has generated its own controversy, with continued debate about the scope of the term [3,4]. Thus debates about the value of genomic and biomarker technologies are often also debates about how to evaluate prognostic assays, and efforts to demonstrate the clinical utility of specific tests are often simultaneously attempts to define clinical utility. Our paper addresses this intersection of ideas and interests in one of the most widely adopted of these technologies: breast cancer prognostics.

Prognostic assays are being positioned as increasingly important tools for physicians to use in the cancer treatment decision-making process [5,6]. These assays are algorithm-based tools that examine a tumor’s gene expression or biomarkers to assess prognosis and, in some cases, predict patients likely response to a specific therapy (for example, adjuvant chemotherapy) [5,7,8]. Currently, these assays are most commonly used in breast cancer prognostics [9,10] with the majority being proprietary products (Oncotype Dx (Exact Sciences), Mammaprint (Agendia), Prosigna (Nanostring), Breast Cancer Index (Biothernostics) and Endopredict (Myriad).

Chemotherapy is a common, yet complex treatment option for breast cancer. This treatment provides effective results for many cancers, but includes significant toxicity and side effects. Due to the myriad factors that influence cancer’s progression, physicians often prescribe chemotherapy to treat breast cancer without definitive knowledge that the chemotherapy will be effective to treat the type of cancer the patient has and therefore may provide no benefit to patients. Research has begun to investigate these issues and has attributed a decrease in the use of unwarranted chemotherapy to the use of “precision medicine” algorithms and assays [11–14]. Prognostic assays purport to reduce overtreatment as they aim to assess the risk of recurrence and response to treatment. For example, research on OncotypeDX – the most commonly used prognostic assay – has reported that the test results altered chemotherapy treatment recommendations in 30–60% of patients [15,16]. As these assays receive reimbursement from public and private payers and are integrated into healthcare services, it has been observed that patients and providers have begun to rely heavily on these results to make decisions about whether or not to undergo chemotherapy [17]. This technology has created a profound shift in cancer care treatment decision-making and processes.

These new tools offer a new conceptualization of value and utility in the cancer care space [18–22]. Recent research has demonstrated that clinical utility of these assays increasingly includes non-traditional factors such as physician confidence, patient anxieties and ‘decision impact’ [23]. Adding the outcome of ‘reducing overtreatment’ as an element of clinical utility for a health technology is a novel idea. When exploring the utility of prognostic assays, attention must be given to the influence of industry on the promotion and positioning of diagnostic and prognostic technologies [1,20,23]. Previous research has highlighted the extensive involvement of industry in producing studies that aim to establish value and demonstrate the clinical utility of these assays and has questioned efforts to generate evidence that might justify the typically high cost of these tools [20,21,23,24]. The clinical utility of a health technology is a key factor for reimbursement decision-making [8,18,20–22,24–28]. For these, often expensive prognostic assays, obtaining reimbursement is critical to integrating them into standard cancer care practices.

Explicating these complex factors of reducing overtreatment, redefining clinical utility and reimbursement are all critical to understanding the value and use of prognostic assays in breast cancer care. Building on a critical body of research that has examined the definition of clinical utility for diagnostic and prognostic tool reimbursement [20,24,25] and evidentiary standards [20,29,30], we further investigate the perceived value and utility of these assays. We also leverage critical scholarship that has investigated the role of industry in defining benefit and promoting their use [20,23,25]. Finally, our study builds on the important work done by diverse scholars [31–34] by extending qualitative inquiry into reducing chemotherapy overtreatment and redefining the clinical utility of diagnostics in breast cancer care.

## Study objective

The objective of this study was to explore key opinion leader’s perspectives on the value, clinical utility, and integration of breast cancer prognostic assays in clinical care.

## Methods

This paper presents data collected during the execution of a larger study on diagnostic innovation. The results presented here build on findings from a scoping review [10] and bibliometric analysis [23].

### Study design

We used semi-structured interviews to address the study objectives and gain insight into key opinion leaders’ perspectives on the use and integration of breast cancer prognostic assays in healthcare services. A qualitative methodology is an ideal approach to explore perceptions and experiences of a phenomenon [35]. The Standards for Reporting Qualitative Research (SRQR) were used to guide the reporting of this study (See S1 File). This study has received ethics approval from the Office of Research Ethics at the University of Toronto (Protocol #00033786). Participants were enrolled after securing written informed consent. Privacy, confidentiality, anonymity, and voluntary participation were also adhered to throughout the study. All authors confirm that all guidelines and regulations were followed correctly and completely.

### Study recruitment & participants

We used a purposive recruitment strategy to identify recent or prolific authors of manuscripts reporting the execution of a decision impact study or manuscripts about decision impact studies. (Decision impact studies propose to evaluate the impact of a medical test or tool on clinical decision-making and have become increasingly prevalent in oncology in recent years, particularly in breast cancer prognostic research). These authors were researchers and physicians/scientists who act as key opinion leader in this field. ‘Key opinion leaders’, ‘opinion leaders’ or ‘thought leaders’ are physicians and researchers who work within healthcare, with industry and are involved in evidence creation and dissemination that advances pharmaceuticals or health technologies such as molecular diagnostics [36–38].

Our goal was to interview 15–20 participants based on feasibility and recommendations from the literature that qualitative studies typically reach data saturation after twelve interviews [39]. The potential interviewees were invited to participate in the study via their publicly available email addresses. Participants were recruited between April 21, 2022 and July 13, 2022. All interested participants provided high-level details about their expertise and knowledge of breast cancer prognostic assays. Our goal was to obtain a diverse sample across types of breast cancer prognostic assays and geographic regions.

### Data collection

The interview guide was developed iteratively by the research team and included questions about prognostic assays, decision impact studies and the clinical utility of the assays. Participants were asked about their experience with prognostic assays and perspectives on their perceived value and clinical utility of these tools. Participants were also asked their perspectives on the purpose, production and use of decision impact studies (See S2 File). Interviews were semi-structured, approximately 1 hour in length and conducted by an experienced interviewer (GP) who had no existing relationship with the participants. Due to the international nature of the study participants, all interviews were conducted virtually via video conferencing. We obtained the participant’s written consent to participate via email, in advance of the interview. Participants were sent a background summary and a sample of the interview questions in advance. Verbal consent for the interview to be video recorded was obtained at the beginning of the interview. Interviews were conducted between May and July 2022. Participants who requested, reviewed their transcripts and/or a draft of this manuscript.

### Data analysis

The interviews were transcribed verbatim and analyzed using thematic analysis [40]. Initially, during the familiarization phase, one researcher (GP) read and coded the transcripts and documented initial *a priori* codes identified through the literature review and new codes that emerged from the data. A second research team members (LS) independently coded a sample of interview transcripts, which were compared against the first team member’s coding of the transcripts. Discrepancies were resolved through consultation within the team. The team developed and refined the codebook iteratively by re-coding and refining *a priori* and emerging themes. Code saturation was reached when no new codes were identified across all transcripts.

## Results

Fifteen participants from nine different countries were included in this study. Participants were researchers and physicians/scientists (oncologists (n = 6), surgeons (n = 5), other (e.g., pathologists, cell biologists) (n = 4)) and had used 4 different breast cancer prognostic assays in their practice and/or research (see participant characteristics in Tables 1 and 2). Participants from this study were drawn from our larger study on diagnostic innovation. Recruitment for the larger study included 90 email invitations of which: 55 did not respond, 12 declined and 23 participated. Of those 23 participants, the 15 breast cancer prognostic assay participants were included in this study.

**Table 1:** Participant nationality.

Nationality	Participant # (n = 15)
USA	4
Canada	3
Australia	2
Mexico	1
Italy	1
India	1
Greece	1
Ireland	1
Brazil	1

**Table 2:** Prognostic assay discussed.

Breast cancer assay (proprietary name)	Participant # (n = 15)
OncotypeDx	9
DCISion RT	3
Endopredict	2
CanAssist Breast	1

The analysis resulted in three themes: 1. role of prognostic assays in identifying overtreatment; 2. integration of assays into clinical use; and 3. jurisdictional variation in use and value. The results are presented according to these themes.

### Role in identifying overtreatment

The majority of participants discussed breast cancer prognostic assays as a tool to support chemotherapy decision-making. Participants detailed the role of chemotherapy in cancer treatment and how these assays could provide guidance regarding the appropriateness of chemotherapy for a particular patient. Breast cancer patients typically have surgery followed by adjuvant therapy, often chemotherapy. Participants acknowledged that standard practice includes sometimes significant overtreatment with chemotherapy. One participant stated: “*I think oncologists are intoxicated with chemotherapy, they feel it will do a lot of benefit, but I think we overestimate, we’ve consistently overestimated the population benefit of cytotoxic chemotherapy” [P210].*

Participants noted that not all patients benefit from adjuvant chemotherapy and that patients should be ‘spared’ this overtreatment, if possible, but determining that which patients should be ‘spared’ had been a challenge until these prognostic assays became available. An oncologist noted “*…if this test is not there, everybody primarily would pick chemotherapy” [P201].* Overwhelmingly, participants described the negative side effects of chemotherapy and the importance of avoiding it if possible. “*…being able to spare women from that possibility of morbidity, mortality, everything along the way, we’re definitely looking at de-escalation whenever possible, and trying to avoid chemotherapy as much as possible”* [P205]. Another participant [P204] stated that at a certain time, all cancer patients in their country were receiving chemotherapy as standard care, but that having an assay available and partially reimbursed changed this practice. He elaborated: “*if chemotherapy can be avoided, in general, patients prefer not to have chemotherapy if they are assured by their physicians that the benefit, they will get from chemotherapy is none or very, very limited” [P204].* A participant described the challenge in reducing overtreatment with chemotherapy:

*The overall benefit, it was only on the order of four and a half percent of patients that would benefit. And yet, I could say easily 70, 80 percent of the women were treated with chemotherapy. So that means a lot of women were receiving it without any benefit. But you couldn’t pick out who they were [before using the assay] [P215].*

Participants also identified value in the support that the assays provided to their clinical recommendation, even if the test suggested the need for chemotherapy treatment:

*If you have a test that reinforces what is happening to you, that is very reassuring to patients. And it does decrease their stress, even if it says, you need chemo. Nobody wants chemo. But if there’s a test that says they really will benefit from it, I think that psychologically helps them tremendously [P216].*

### Integration into clinical practice

Our participants provided insights into the deliberate integration of these assays into clinical practice and how these assays are changing care pathways regarding cornerstone cancer treatments. Participants identified the value of the assays as improved quality of life and reduction in overtreatment, often as a justification for their high cost. These assays are expensive with use dependent on insurance coverage in the majority of cases. Participants detailed how the definitive result and removal of downstream health system use is positioned to integrate the tools and justify the high costs for these assays: “*…we have done a study just to show to the payer that if we could do the genomic test, in particular, Oncotype, we can save a lot of money, but this is not the principal problem, we can save lots of trouble to the patient because we can save a lot of chemotherapy” [P202].* Some participants noted that when the cost was factored into the total, overall costs were still reduced as chemotherapy was no longer required, which lead to additional downstream healthcare savings:

*So certainly, by doing this test, the cost savings are humongous. The cost of chemotherapy in private setups can be like at least four or five times the cost of this test. So there is definite personal savings for any private patient. And this is even more enhanced at the government level. So there the savings are for thousands of patients [P201].*

Conversely, other participants discussed how the high cost rendered the assays prohibitive. A participant discussed how chemotherapy drugs are getting cheaper, so the high price of these assays is less justifiable: *So, I think the economic analysis is very contingent on the cost of the drugs… chemotherapy is becoming cheaper… So as the drugs become less expensive, then it becomes an issue [P210].*

### Jurisdictional variation in perspectives and use

Participants discussed how cancer care is perceived and practiced differently in different regions and countries. Historical uses of cancer treatments have differed, particularly between USA and Europe. Traditionally, providers in the US have used chemotherapy more commonly, while providers in Europe have been more judicious. Participants described perspectives and practices regarding chemotherapy that varied greatly and appeared to be rooted in this UK vs US training paradigm. *“… the attitude to chemotherapy is quite different [in Australia] in that our thinking is somewhat between US and UK/Europe, in that, we have never been as pro-chemo as, you know, the US guidelines” [P203*]. Another participant stated: *“Most of the Greek medical oncologists are trained in the States, not in England…Traditionally, in UK, they were in favour of hormonal therapies and in the United States in favour of chemotherapies” [P204].*

Participants discussed that providers and reimbursors view healthcare as a local phenomenon and therefore place significant weight on local or jurisdictional evidence and practices:

*…it’s really interesting, how much healthcare is still very much of a local phenomenon... in the early days and we spoke to a lot of Canadian oncologists, and they would almost kind of look at the results from the US [and say], we’re not like that. We don’t do things like that… [P215].*

*So I guess for decision makers, Mexican patients were not included, but we did another study here and it’s the same. We’re not different [P220].*

Participants also discussed that countries and cultures who view themselves as ‘different’ viewed the benefits and value of the prognostic assays differently as well. Participants discussed that these varied perspectives produce uneven interpretations of value for the assays:

*…countries in Western Europe and in Scandinavia. They still don’t pay for the test. And so, because there’s still a fundamental belief that well, we’re different, we actually have very good pathologists, we actually, we don’t think the value is there for our society of physicians and pathologists [P215].*

*The pathologists have sort of influenced the discussion here and said, in the vast majority of cases, you don’t actually need to have this assay. So, the issue in Australia is [the assays have] not been funded, because they don’t know how to select the patients that need it, they’re unable to prove that there is a cost effectiveness by introducing the assay, they can’t see any benefit in terms of health savings [P221].*

## Discussion

In this study we aimed to explore key opinion leader’s perspectives on the integration of and use of prognostic assays in breast cancer care. Our results illuminate the impact of the positioning of this technology on identifying overtreatment, standard care processes, and the jurisdictional variation in perceived value and use of these assays.

Research has identified that breast cancer is currently often overtreated [14,34,41]. Providers have begun to address this issue and prognostic assays have become prevalent tools in efforts to identify inappropriate chemotherapy use. The integration of these assays into standard practice presents clinical and ethical shifts for providers as these assays redefine cornerstone cancer treatments and treatment decision-making. This shift contravenes the “imperative to treat” [34] often felt by patients and providers [9,34]. Our participants identified reducing overtreatment as the primary purpose of these assays. They described the impact of believing chemotherapy was not the best treatment option, but, without more information, and being influenced by their training, they overtreated patients as standard practice. Our findings align with Ross and colleagues who observed that prognostic assays “appeared to make a rejection of the treatment imperative an acceptable choice, due to its positioning as a ‘scientific’, ‘advanced’ and ‘state of the art’ technique which reduced uncertainty” [34]. While some studies have reported that oncologist have felt uneasy about recommending against chemotherapy, because they practice in the “context of a ‘dose culture’” and are “trained to believe in chemotherapy” [9]; our participants reported that the assays provided definitive results which provided confidence – for both patients and providers – in deciding whether to undergo chemotherapy treatment.

Our participants provided rich data on the emerging challenge created by a tool that reduces downstream overtreatment, while potentially creating a new issue of over testing. Integrating these assays into standard care therefore reducing overtreatment with toxic drugs, but increasing laboratory testing creates a unique situation for providers and patients to navigate. Bombard and colleagues (2015) raise this issue in their study of the use of gene expression profiling and note that indiscriminate use of these assays is not only costly, but contributes to the significant health system issue of overuse of laboratory testing [32]. This issue necessitates a weighing of benefits, harms and patient outcomes. The authors call for clinical practice guidelines to standardize genomic test ordering – to mitigate potential laboratory overuse while realizing the benefits of reducing unnecessary chemotherapy [32]. While participants in our study did not raise the issue of over testing – emphasis was placed on the negatives of side effects and the significant benefits of not inappropriately treating with chemotherapy – this emerging issue necessitates further investigation.

Our findings further illustrate the complexity of determining clinical utility for novel diagnostics [20–22,24,26]. The participant’s perspectives on chemotherapy overuse, and the role of assays to address this, highlights that the assays have been framed as an integral tool to address the problem of overtreatment. In doing so, these assays add a novel patient-focused outcome that exists outside the traditional scope of clinical utility outcomes. The participants conveyed this extended definition of clinical utility – identifying overtreatment – as the key purpose of the tests. Our recent research has demonstrated that the integration of these new ideas has been significantly supported by evidence creation and marketing done by the producers of the assays [23]. All of our participants had conducted research on the ‘decision impact’ of these assays and expressed this extended definition of clinical utility. These perspectives reinforce that these assays are advancing the novel role of prognostic assays to identify overtreatment, thereby extending boundaries of clinical utility in this space. Our earlier scholarship has critiqued this elaboration of clinical utility [1,20,23], the uncertainty around the evidentiary standards [1,20,21,24] and industry’s role in these activities [20,23]. Industry developing and promoting new technologies (and required testing processes) to address overtreatment adds financial and commercial factors into the treatment decision-making process.

The cost of the assays and varied reimbursement schemes were a significant area of discussion. A number of participants who thought the high cost was justified and had conducted a cost analysis, reported that while the assay is expensive, the downstream savings, with patient outcomes and healthcare savings, justified the cost. On the contrary, other participants describe the cost as prohibitive and argued that currently chemotherapy drugs are generic and these costs are low relative to the cost of the assay. These disparate perspectives reflect the vastly different health systems within which providers practice. If the assays were fully or partially reimbursed had significant impact on the costs of the assays and subsequently their perceived value in their local context. This disparity sometimes lead to a ‘physician’s paradox’, continuing to overtreat patients because the assay was not available or too expensive. The gravity of the issue – severity of overtreatment – also varied depending on the system within which care is provided. Within these contexts the issue of price can become an epilogue to broader systemic issues that create scenarios where overtreating a patient is the cheaper option because the high cost of a prognostic assay.

The impact of the health system on costs and subsequent use leads to a novel and unexpected theme in our findings. The participants discussed jurisdictional variation in perceived value and use for cancer treatment and how this adds to the complexity of these new conceptualizations of overtreatment, clinical utility, and standard care. The perspectives described illuminate the subjectivity of cancer treatment and the contested nature of the space within which these assays attempt to add value. While overtreatment in breast cancer is occurring all over the world, our international participants shed light on the geographic variability of overtreatment and the important implications this variability has for the value add of these assays. Participants describe a “US/UK school of thought” dichotomy where US training is described as using chemotherapy more liberally, and UK training using endocrine therapy more frequently. While our participants were from nine different countries, this dichotomy in training was referenced frequently and participants spoke of how Latin and South American countries followed the US paradigm; while Australia and most European countries aligned with the UK school of thought. These varied approaches to cancer treatment, and varied use of chemotherapy as the dominant cancer treatment created disparate perceptions of value for the assays. In countries where chemotherapy was used more judiciously, therefore overtreatment with chemotherapy was less, the perceived value of the assay was not high. In some countries, such as Brazil, where overuse of chemotherapy has been reported to be as high as 60% [42]; the potential to reduce overtreatment with chemotherapy was significant. While research has begun to explore jurisdictional variation in cancer diagnosis, treatment and specifically chemotherapy use [43–46], much work needs to be done in this area. Our study extends this analysis to highlight the role of entrenched jurisdictional perspectives on cancer and treatment on the value of the assay.

### Directions for future research

Our findings call for further research to understand the implications of the uptick in expensive, proprietary testing to purportedly reduce overuse of adjuvant chemotherapy. Significant changes in care pathways such as these often have numerous intended and unintended consequences that require careful consideration and evaluation.

### Strengths and limitations

The strengths of this study are the heterogenous sample: we interviewed researcher-physician/scientists from numerous countries, who worked in different type of health systems and had experience with a variety of prognostic assays. The limitations of this study are the recruitment method: we recruited through email only and we did not include patients or payers in our sample. Also, participants were participating in a larger study about decision impact studies for prognostic assays and all were authors of decision impact studies. Our previous work showed that these studies were often produced by industry, therefore our participants were potentially not a non-biased group, although perspectives expressed represented a broad spectrum of views.

## Conclusion

The results of this study provide insights into the integration of prognostic assays into clinical decision-making processes. These assays are extending the boundaries of their clinical utility through identifying overtreatment and unnecessary care. Efforts to integrate these assays and justify their high prices are explored. Finally, these results illuminate the subjectivity of cancer treatment and the contested space within which these tools attempt to add value.

## Supporting information

S1 File SRQR reporting checklist. (PDF)

S2 File Interview guide. (PDF)
