# Supplementary material for: Prognosis, treatment decision-making and value: A qualitative exploration of the emerging role of breast cancer prognostic assays
Source: PLoS One. 2025 Jul 18;20(7):e0322509. doi: 10.1371/journal.pone.0322509 (PMC12274007; doi:10.1371/journal.pone.0322509)
Supplement: S2 File — (PDF) [file pone.0322509.s002.pdf]

Diagnostic Innovation Study –Interview Guide

Preamble

Thank you for taking the time to speak with me today. As you know from the email we sent you, this study explores the origins, understandings, purpose and use of decision impact studies (DIS) in genomic medicine in cancer care. We would like to learn about your experiences with prognostic assays and DIS and your perspectives of the perceived value and use, opportunities and challenges and how it is situated in the process of advancing genomic medicine. We have conducted a scoping review and bibliometric analysis of decision impact studies to inform our knowledge and understanding of the field. You were recognized as a contributor to this literature.

Intro/opening question

1. Please tell me about your research/work? *(Variable – breast cancer or other)*

Prognostic Assays

2. Please tell me about your experience with prognostic assays. Their use, perceived value and how they are situated in the process of advancing cancer care.

Your experience with DIS

3. We are interested in getting a sense of researcher-physician/scientist's understanding of and experience with DIS. Why these studies are conducted and framed in this way, how familiar researchers are with DIS as a purposely created type of research, the origins of this type of study, the use and validation of this type of study.

Clinical Utility

4. What is your definition of clinical utility for a prognostic assay? What role do DIS play in providing evidence of CU?
5. Talk about your perceptions/understanding/challenges with CU in your field?
6. Can you speak about prognostic and predictive results? The importance of this distinction?

Purpose

7. Please tell me about the role of DIS in: Treatment decision-making? Provider confidence? Patient confidence?
8. Please tell me about the impact on physician autonomy?

Methodology

9. Regarding new technologies, genomic innovation in this case, there is a call for more rigorous studies, more prospective studies, how do DIS fit into this call?

Evidence

10. Intended impact of evidence produced for insurance re-imbursement purposes?

11. Intended impact of evidence produced for CPG inclusion?
12. Intended impact of evidence produced for regulatory approval?

Final thoughts

13. Is there anything else we haven't talked about that would give us a better understanding of prognostic assays and decision impact studies?
