# Supplementary material for: Prognosis, treatment decision-making and value: A qualitative exploration of the emerging role of breast cancer prognostic assays
Source: PLoS One. 2025 Jul 18;20(7):e0322509. doi: 10.1371/journal.pone.0322509 (PMC12274007; doi:10.1371/journal.pone.0322509)
Supplement: Attachment — Submitted filename: Response to Reviewers - Parker et al. .docx [file pone.0322509.s004.docx]

Dear Dr. Chenette,

Thank you for the invitation to revise and resubmit our manuscript. We have addressed the Editor’s and Reviewer’s comments and believe that incorporating their feedback has enhanced the quality of our paper. Their comments were insightful, and we believe this review process has resulted in an improved manuscript. A detailed response to the comments may be found in the table below.

Sincerely,

Gillian Parker

| **Editor Comment** | **Action** |
| --- | --- |
| Please ensure that your manuscript meets PLOS ONE's style requirements, including those for file naming | We have:  -Updated the title page formatting as per the provided title page guidance document  -Renamed the Supplementary file to: S1 – SRQR Reporting Checklist |
| Please state what role the funders took in the study. | We have added the following sentence in the funding statement:  *The funders had no role in study design, data collection and analysis, decision to publish, or preparation of the manuscript.* |
| Please confirm at this time whether or not your submission contains all raw data required to replicate the results of your study. | We have added the following data availability statement:  *Data Availability Statement: The raw data for this study are transcripts of interviews, which contain potentially identifying participant information. Relevant, de-identified excerpts of the transcripts are included in the paper. For external requests or inquiries regarding data access, please contact the corresponding author. Requests will be reviewed to ensure they meet ethical and data governance guidelines.* |
| **Reviewer Comment** | **Action** |
| **Methods** | |
| Some participants were family physicians/general practitioners, who are not typically involved in requesting, processing, and interpreting breast cancer prognostic assays. What was the rationale for including these participants? | Thank you for highlighting this. We have corrected the role, added counts for the participants, and updated references in the manuscript. |
| Information on participants’ practice specialty should also be included in Table 1. | Thank you for this suggestion. We have added this information to the results section. It is disaggregated from Table 1 to support anonymity. |
| The questions explored in the semi-structured interviews should be described in more detail. | Detail has been added to the data collection section of the manuscript. In addition, the interview guide has been added as Supplementary File 2. |
| Ideally, the authors should provide the initial interview guide as part of the supplementary material. | We have added the Interview Guide as Supplementary File 2. |
| **Results** | |
| Although most participants discussed prognostic assays supported by evidence for a predictive role (Oncotype Dx and DCISionRT), some mentioned assays lacking strong evidence for predicting treatment benefit in addition to their prognostic role. Did the authors observe any differences in perspectives based on the specific assay discussed?  Were there any themes related to clinician preference for tools with both prognostic and predictive value, especially in settings where more than one assay is available? | Thank you for these thoughtful questions. They offer important inquiry for future research!  We did not observe any significant or reportable themes regarding differences in perspectives based on the specific assay discussed.  The topic of clinician preference for specific tools was not included in the interview questions and we did not observe these themes in the data. |
| **Discussion** | |
| According to the results, the primary perceived role of breast cancer prognostic assays is to reduce overtreatment. How does this relate to reimbursement decisions in some jurisdictions for patients with smaller tumors (e.g. T1b, node-negative cancers), since these patients are much less likely to receive chemotherapy in the absence of a prognostic assay?  Were there any discussions about the use of these assays to escalate treatment for some patients? | As stated above, we appreciate these insightful questions and agree they are important topics to be explored in future research.  The theme of reducing overtreatment emerged in the interview data.  The topic of reimbursement decisions regarding smaller tumors was not included in the interview questions and we did not observe significant or reportable themes on this topic.  We did not observe themes regarding the use of assays to escalate treatment plans. |
| **Limitations** | |
| Could the authors clarify why conducting interviews virtually is considered a limitation in this study?  In addition to the lack of patient perspectives, the absence of payer perspectives may also represent a limitation, given the manuscript’s strong focus on value, cost, and reimbursement decisions. | Thank you for this note. Upon reflection, virtual interviews were a strength as they facilitated the geographically diverse participant sample. We have removed this text.  Thank you for the suggestion. We have added lack of payer participation to the limitations. |
